# The complete mitochondrial genome of *Muscina pascuorum* (Diptera: Muscidae)

**DOI:** 10.1080/23802359.2020.1848473

**Published:** 2021-01-13

**Authors:** Jingjing Huang, Zixiang Ni, Hua Wang, Li Zhang, Jie Yan, Shengbin Bai

**Affiliations:** a Department of Forensic Science, School of Basic Medical Sciences, Xinjiang Medical University, Urumqi, Xinjiang, China; b Department of Forensic Science, School of Basic Medical Sciences, Central South University, Changsha, Hunan, China; c Department of Histology and Embryology, School of Basic Medical Sciences, Xinjiang Medical University, Urumqi, Xinjiang, China

**Keywords:** Mitochondrial genome, *Muscina pascuorum*, phylogenetic relationships

## Abstract

*Muscina pascuorum* (Diptera: Muscidae) represents an important hygiene pest. The complete mitochondrial genome (mitogenome) of *M. pascuorum* was first sequenced and annotated in this study. The full length of mitogenome was 14, 940 bp, consisting of 13 protein-coding genes (PCGs), two ribosomal RNA (rRNA), 22 transfer RNA (tRNA), and one AT-rich region. The nucleotide content of these flies was 40.0% A, 13.2% C, 9.1% G, and 37.6% T. This study illustrates that the arrangement of the genes was identical to classical metazoans. Besides, the phylogenetic analyses indicated that the branch of *M. pascuorum* was clustered separately from the common three *Muscina spp* in the tree. This genome provides an essential reference for understanding the phylogenetic relationships of Muscidae.

The Muscidae is of great significance in hygienic pests, covering almost the whole world (Ren et al. 2018). *Muscina pascuorum* Meigen, 1826, belonging to Muscidae family and Diptera order, is usually found entirely in late summer and autumn (Johnson 1926). The adults tend to be live in places with cover soft fungus-like swamps, puddle, and lawn (Curran 1926). Compared with single genes or subregions of the mitochondrial genomes (mitogenomes), mitogenomes have remarkably improved the accuracy of taxonomic relationships and the efficacy of phylogenetic analyses, especially for closely related species (Nadimi et al. 2016). In this study, the full length of mitogenome was 14,940 bp (GenBank accession no. MT017715), consisting of 13 protein-coding genes (PCGs), two ribosomal RNA (rRNA), 22 transfer RNA (tRNA), and one AT-rich region. The nucleotide content of these flies was 40.0% A, 13.2% C, 9.1% G, and 37.6% T.

Adult specimens of *M. pascuorum* were captured in Ürümqi city (43°50′N, 87°37′E), Xinjiang province, China, in June 2019. These species were identified by a morphological expert and then preserved at −80 °C in Guo’s lab (Changsha, China) with a unique code (CSU19111968). The DNA was extracted from thoracic tissues of the flies by the QIANamp Micro DNA Kit (Qiagen Biotech Co., Ltd., Shanghai, China). Sequences were performed on an Illumina HiSeq 2500 Platform. Mitochondrial de novo assembly was performed using MITObim v 1.9 and SOAPdenovo v2.04 (https://github.com/chrishah/MITObim and http://soap.genomics.org.cn/soapdenovo.html). Finally, the rough boundaries of each gene were initially identified using MITOS2 Web Server (http://mitos2.bioinf.uni-leipzig.de/index.py) (Ren et al. 2020).

Phylogenetic analysis included *M. pascuorum* and 11 Muscidae species using maximum likelihood (ML) method based on 13 PCGs and *Megaselia scalaris* as the outgroup (Figure 1). ML analysis was carried out with IQ-TREE v1.6.12 (Ren et al. 2020). The tree showed that *M. pascuorum* belongs to the *Muscina* subgenus, which was clearly separated from the clade of Muscidae species. This study provided several crucial genetic data of dipteran mitogenomes for further study on evolution analysis and species identification.

**Figure 1.**
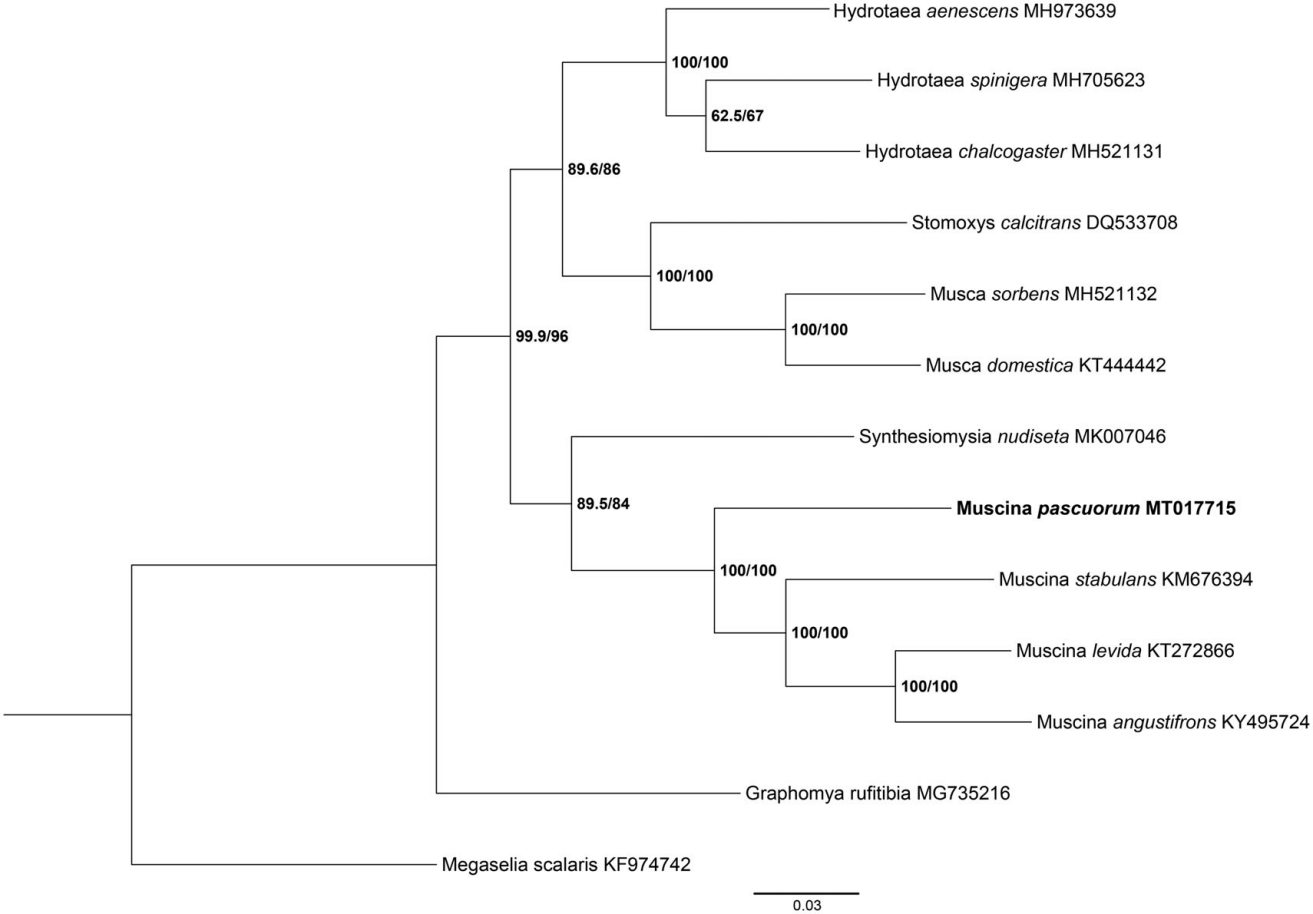
Phylogenetic analysis of *M. pascuorum* with 11 Muscidae species was generated by using maximum likelihood (ML) method. *Megaselia scalaris* was referred to as the outgroup.

## Data Availability

The raw data that support the findings of this study are openly available in the SRA (Sequence Read Archive) database of NCBI (National Center for Biotechnology Information) via accession numbers at https://www.ncbi.nlm.nih.gov/sra/?term=SRR12587884 (SRR12587884). The assembled mitochondrial genome is available on NCBI at https://www.ncbi.nlm.nih.gov (accession No. MT017715).
